# Syndromic Congenital Heart Disease Diagnosed in Adulthood: A Reminder of the Phenotypic Variability of Alagille Syndrome

**DOI:** 10.1155/cric/5884548

**Published:** 2026-01-09

**Authors:** Matthew K. Campbell, Jessica Hallam, Samuel L. Casella, Sangeeta Shah

**Affiliations:** ^1^ Department of Internal Medicine, VCU Health, Richmond, Virginia, USA; ^2^ Department of Internal Medicine, Division of Cardiology, VCU Health Pauley Heart Center, Richmond, Virginia, USA; ^3^ Department of Pediatric Cardiology, Children′s Hospital of Richmond at VCU Health, Richmond, Virginia, USA

**Keywords:** adult congenital heart disease, Alagille syndrome, JAG1, peripheral pulmonary stenosis, variable expressivity

## Abstract

Alagille syndrome is a rare multisystemic genetic condition most commonly associated with neonatal liver disease. Variable expressivity is a defining feature of Alagille syndrome, resulting in a broad spectrum of phenotypic variation among individuals who meet the diagnostic criteria. We present an atypical case of cardiac‐predominant Alagille syndrome diagnosed in adulthood after the detection of peripheral pulmonary stenosis on cardiac magnetic resonance imaging (CMR).

## 1. Introduction

Alagille syndrome is an autosomal dominant condition estimated to be present in 1:30,000 live births [1]. Neonatal liver disease is the hallmark of Alagille syndrome, and recent data suggest that only 40% of patients will survive to adulthood with their native livers [1]. There are, however, a broad spectrum of associated extrahepatic manifestations. These are most notable for distinct ophthalmologic and vertebral defects, characteristic facial features, and a multitude of cardiovascular anomalies, which, in addition to cholestasis, comprise the five major clinical features. The majority of individuals with Alagille syndrome are diagnosed within the first year of life due to early‐onset liver disease; however, the high degree of variable expressivity characteristic of Alagille syndrome can make the diagnosis of a primarily extrahepatic phenotype challenging [1–3]. Here, we will illustrate the diagnostic journey of an adult presenting with nonspecific cardiopulmonary symptoms, ultimately leading to genetic confirmation of Alagille syndrome.

## 2. Case Presentation

**Figure 1:**
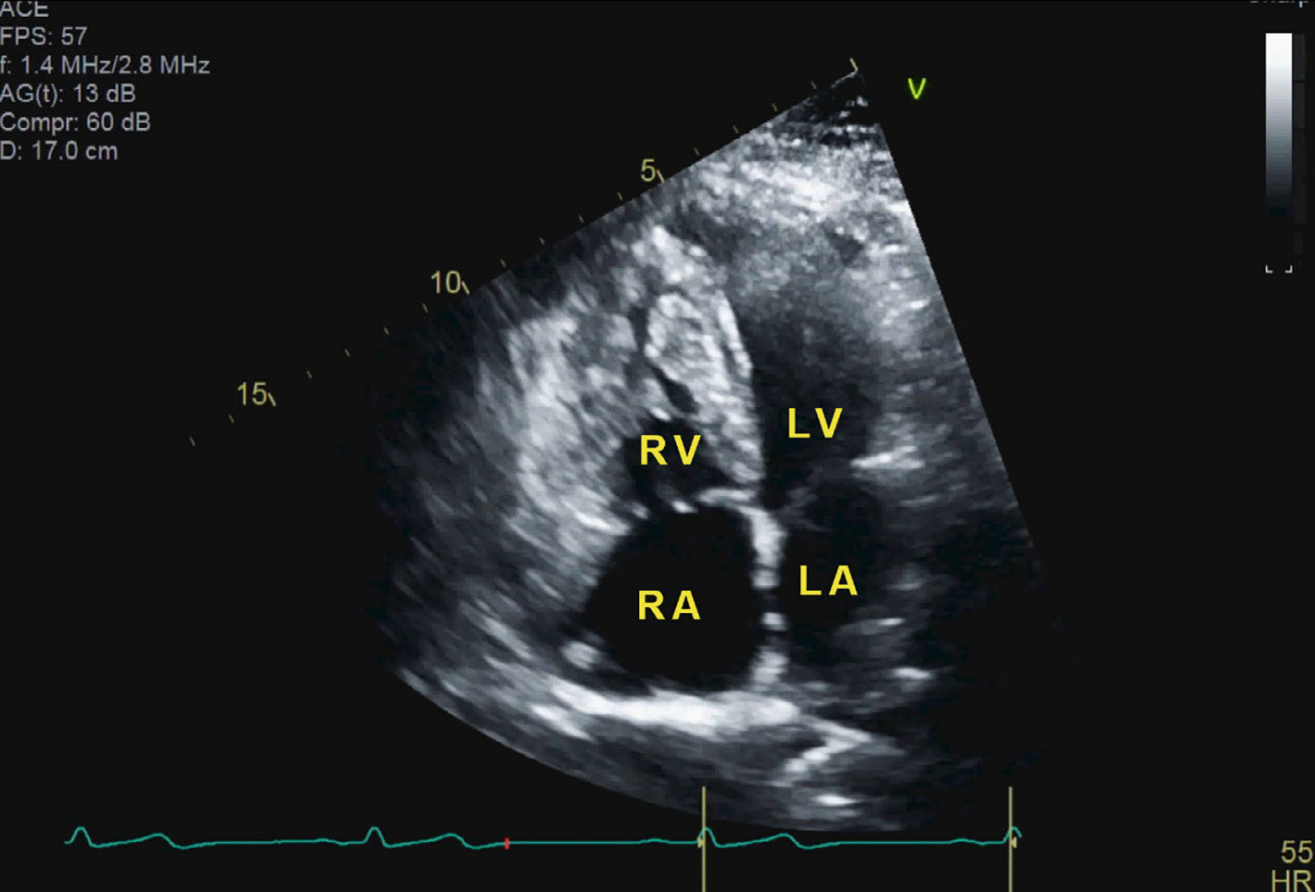
Transthoracic echocardiogram. Four‐chamber view demonstrating right ventricular hypertrophy. Abbreviations: RA, right atrium; RV, right ventricle; LA, left atrium; LV, left ventricle.

**Figure 2:**
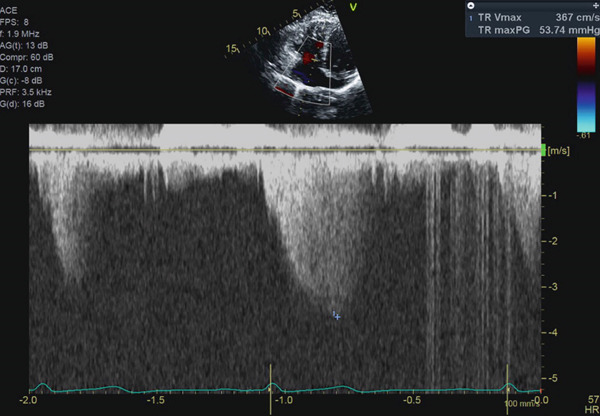
Transthoracic echocardiogram. Apical four‐chamber view. Continuous wave Doppler estimating an elevated tricuspid regurgitation peak gradient (TRPG) at 54 mmHg, suggestive of increased pulmonary artery pressure.

A 46‐year‐old woman with a history of hypertension, a persistent left superior vena cava, a perimembranous ventricular septal defect (VSD), and a prior diagnosis of idiopathic intracranial hypertension presents to cardiology clinic for several years of worsening exertional dyspnea. Physical examination is notable for a Grade II/VI systolic murmur in the precordium and a loud S2. Height is measured at 1.6 m. Laboratory evaluation reveals nonproteinuric renal dysfunction with an eGFR of 55 mL/min/1.73 m^2^. B‐type natriuretic peptide is within normal limits. Electrocardiogram shows normal sinus rhythm with an incomplete right bundle branch block. Transthoracic echocardiogram (TTE) redemonstrates a small perimembranous VSD partially occluded by aneurysmal tissue of the tricuspid subvalvular chordae (Supporting Information 1: Video S1). The TTE also reveals right ventricular hypertrophy (Figure 1) and an elevated tricuspid regurgitation peak gradient (TRPG) of 54 mmHg (Figure 2), suggestive of increased pulmonary artery pressure.

**Figure 3:**
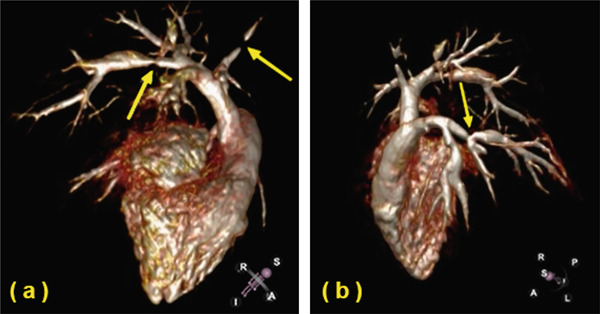
Cardiac MR angiography visualizing several areas of focal stenosis in (a) the right pulmonary artery and (b) the left pulmonary artery with specific orientations as depicted in the bottom right corners. Abbreviations: S, superior; I, inferior; A, anterior; P, posterior; R, right; L, left.

She is referred for left and right heart catheterization. Coronary angiography is negative for significant atherosclerotic disease. Right ventricular systolic pressure (RVSP) is elevated at 75 mmHg, and mean pulmonary artery pressure (mPAP) is elevated at 33 mmHg. Pulmonary capillary wedge pressure is 7 mmHg, resulting in a transpulmonary gradient of 26 mmHg. Pulmonary vascular resistance is elevated at 6 Wood units. She is diagnosed with precapillary pulmonary hypertension. Tadalafil 40 mg daily and ambrisentan 5 mg daily are sequentially initiated with minimal improvement in symptoms. CMR is obtained to assess the hemodynamic significance of her VSD. CMR shows no significant shunt through the VSD (QP:QS ratio of 1:1) but does demonstrate several areas of previously unidentified stenosis in the bilateral pulmonary arteries (Figure 3 and Supporting Information 2: Video S2). She is referred to an adult congenital heart disease (ACHD) specialist.

At her initial ACHD clinic visit, she is noted to have a previously unrecognized systolic ejection murmur over the right shoulder, as well as low‐set ears, deep‐set eyes, a prominent chin, and short thumbs. These exam findings in combination with the recently discovered pulmonary stenoses prompt concern for an underlying genetic syndrome. Noonan syndrome, Alagille syndrome, and Williams syndrome are felt to be the most likely differential diagnoses. She is referred to clinical genetics and has genetic testing of 55 genes performed using the INVITAE Congenital Heart Disease Panel. While awaiting results, she undergoes computed tomography angiography (CTA) of the chest to further characterize the stenoses noted on CMR. The CTA reveals multifocal branch stenosis with areas of poststenotic dilatation, confirming peripheral pulmonary stenosis. Several days later, the gene panel results with a pathogenic loss of function mutation in Exon 15 of the JAG1 gene (c.1984del (p.Ala662Profs∗81)), consistent with a diagnosis of Alagille syndrome.

Following her diagnosis, our patient undergoes a thorough hepatology evaluation; she did report a history of undifferentiated neonatal jaundice. Liver ultrasound reveals normal morphology and homogenous echotexture without evidence of steatosis or fibrosis. Laboratory evaluation is notable for mild chronic thrombocytopenia; however, her transaminases, bilirubin, and INR are notably within normal limits. She does not undergo liver biopsy. She has no evidence of butterfly vertebrae on chest radiographs, nor any other clinically significant skeletal abnormalities. She had followed with ophthalmology for several years prior to her diagnosis for a subretinal scar as well as anatomically narrow angles for which she had undergone bilateral laser peripheral iridotomy. She does not have posterior embryotoxon.

After discussion in a multidisciplinary conference, she is referred to a congenital interventional cardiologist for pulmonary artery rehabilitation. She undergoes four catheterizations for extensive pulmonary artery dilations, recanalization of atretic branches, and stenting, with gradual improvement in her RVSP and mPAP to 42 and 24 mmHg, respectively. She continues to follow in the ACHD clinic and is being closely monitored for progressive stenoses or restenosis of the previously dilated vessels using a combination of echocardiography, lung perfusion scans, and serial axial imaging.

Familial testing confirms a diagnosis of Alagille syndrome in one of her three children when her daughter tests positive for the same pathogenic JAG1 variant at the age of 18. She had carried the diagnosis of branch pulmonary artery stenosis for several years prior to her mother′s diagnosis, and she had known hepatic abnormalities with elevated transaminases and abnormal echotexture of the liver. She has normal renal function and no evidence of butterfly vertebrae; however, similar to her mother, she also has anatomically narrow ocular angles. She has characteristic facial features of Alagille syndrome with a prominent forehead and a pointed chin. She adheres to regular echocardiographic surveillance and has undergone cardiopulmonary exercise testing, lung perfusion imaging, and invasive hemodynamic monitoring to characterize her anatomy and assess cardiac function. To date, she has not required pulmonary artery dilation or stenting. She continues to follow with the same team of specialized providers who care for her mother. While she has yet to desire pregnancy, genetic counseling is accessible for further education on the one in two risk of passing along the shared pathogenic JAG1 variant to future generations.

## 3. Discussion

**Figure 4:**
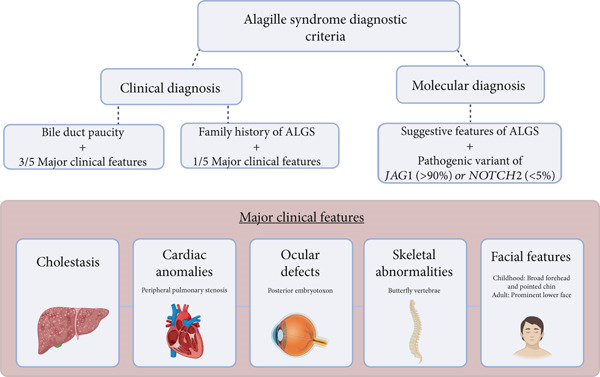
Graphic depiction of the clinical and molecular diagnostic criteria for Alagille syndrome. Abbreviation: ALGS, Alagille syndrome.

Cardiac manifestations are estimated to be present in over 90% of patients with Alagille syndrome and are the second most common clinical feature, surpassed only by the incidence of liver disease [1, 4]. There is a broad spectrum of cardiovascular defects associated with Alagille syndrome; however, peripheral pulmonary stenosis, as seen in our patient, is the most frequently reported [4]. The overall clinical presentation of patients with Alagille syndrome is known to vary widely between family members, and drastically different phenotypes have even been reported between monozygotic twins sharing the same genetic mutation [5–7]. To account for this variability, both clinical and molecular diagnostic criteria have been defined (Figure 4) [8]. There have been reports of families with distinct JAG1 variants and a spectrum of cardiac defects in which no individual met diagnostic criteria, suggesting a spectrum of disease associated with JAG1 mutations that exists both within and outside a formal diagnosis of Alagille syndrome [9]. Although not part of the current diagnostic criteria, renal disease, most often in the form of renal dysplasia, is common in patients with Alagille syndrome [8]. Similarly, while not required for diagnosis, noncardiac vascular anomalies including intra‐ and extracranial aneurysms, coarctations of the aortic and renal vasculature, and even rare cases of moyamoya disease have been described with a combined estimated prevalence between 15% and 30% [8, 10, 11]. While less common than many of the clinical manifestations included in the diagnostic criteria, these noncardiac vascular anomalies are estimated to contribute to up to a third of the mortality in patients with Alagille syndrome [8, 10, 11].

This case adds to the existing literature by describing an atypical cardiac‐predominant presentation of Alagille syndrome not diagnosed until the fifth decade of life. Unlike most patients who are diagnosed in infancy due to severe cholestasis, our patient′s reported jaundice self‐resolved. While transient hyperbilirubinemia can be seen for many reasons in the neonatal population, we can assume her underlying genetic condition played a role. At the time, her self‐limited hepatic manifestations conferred an increased probability of transplant‐free survival compared to those with persistent cholestasis. That being said, patients with more severe forms of liver disease likely have a higher chance of being accurately diagnosed with Alagille syndrome early in life since their phenotype is more classically associated with the disease process.

As our patient grew older and was no longer viewed through the lens of a young child with cholestasis, a diagnosis of Alagille syndrome became more elusive. Throughout childhood, she was found to have a VSD, a persistent left superior vena cava, and was noted to have evidence of developmental delay. In adulthood, she was diagnosed with idiopathic intracranial hypertension, renal dysfunction of unclear etiology, and bilateral ocular defects requiring intervention. Retrospectively, it seems clear to associate this constellation of signs and symptoms with Alagille syndrome; however, in the absence of significant hepatic dysfunction or lifestyle‐limiting cardiopulmonary symptoms, there had never been a high enough index of clinical suspicion to seek a unifying diagnosis.

It was not until symptoms from one of her underlying manifestations worsened that there was an impetus to pursue further diagnostic testing. The CMR was ordered to evaluate the hemodynamic significance of her VSD, which, at the time, was thought to be the most likely etiology for her precapillary pulmonary hypertension. However, the CMR quickly became the inflection point of her diagnostic journey when it revealed the first evidence of pulmonary artery stenosis. It was the identification of this previously occult cardiovascular anomaly that ultimately led to specialist referral and genetic testing to seal her diagnosis. With proper localization of the defects, her treatment strategy was able to shift from standard pulmonary hypertension medications to an individualized procedural approach to address the stenotic vessels themselves. While CMR remains a limited resource, the presence of a systolic murmur upon auscultation of the posterior lung fields, as was present in our patient, should prompt clinicians to consider the possibility of pulmonary artery stenosis.

This case highlights the importance of general practitioners and cardiologists alike having familiarity with the signs and symptoms of syndromic heart disease and, when unsure about the next steps, having a low threshold to refer to a specialist for assistance establishing a definitive diagnosis and initiating familial cascade testing. As demonstrated by our patient and her daughter, such a diagnosis often impacts the lives of both the proband and their loved ones. Preimplantation genetic testing is an option for affected family members who wish to reduce the risk of passing on such an inherited condition to their offspring. The decision to proceed with embryo testing is patient‐specific, and the preceding discussion should be facilitated by a professional genetic counselor.

Given the phenotypic variability of patients with Alagille syndrome, especially those diagnosed in adulthood, the importance of increasing awareness of the condition itself cannot be overstated [12]. By continuing to disseminate case reports and series of atypical presentations of Alagille syndrome, we can work toward increasing recognition and improving the time to diagnosis.

## Consent

No written consent has been obtained from the patient, as there is no patient‐identifiable data included in this case report.

## Conflicts of Interest

The authors declare no conflicts of interest.

## Funding

No funding was received for this manuscript.

## Supporting Information

Additional supporting information can be found online in the Supporting Information section.

## Supporting information


**Supporting Information 1** Video S1: Transthoracic echocardiogram. Five‐chamber view with color Doppler showing a small perimembranous VSD partially occluded by aneurysmal tissue near the right coronary cusp.

**Supporting Information 2** Video S2: Cardiac MR angiography revealing multifocal areas of stenosis in the bilateral pulmonary arteries.

## Data Availability

Data sharing is not applicable to this article as no new data were created or analyzed in this study.
